# Comprehensive analysis of karyopherin alpha family expression in lung adenocarcinoma: Association with prognostic value and immune homeostasis

**DOI:** 10.3389/fgene.2022.956314

**Published:** 2022-08-03

**Authors:** Xiuwen Lan, Lin Zhao, Jian Zhang, Yingchun Shao, Yunmeng Qv, Jian Huang, Li Cai

**Affiliations:** ^1^ Department of Critical Care Medicine, Harbin Medical University Cancer Hospital, Harbin, China; ^2^ The Fourth Department of Medical Oncology, Harbin Medical University Cancer Hospital, Harbin, China; ^3^ Department of Thoracic Surgery, Harbin Medical University Cancer Hospital, Harbin, China; ^4^ Department of Pharmacology, College of Pharmacy, Harbin Medical University, Harbin, China

**Keywords:** lung adenocarcinoma, the KPNA family, immune homeostasis, biomarker, potential target

## Abstract

**Background:** Karyopherin alpha (KPNA), a nuclear transporter, has been implicated in the development as well as the progression of many types of malignancies. Immune homeostasis is a multilevel system which regulated by multiple factors. However, the functional significance of the KPNA family in the pathogenesis of lung adenocarcinoma (LUAD) and the impact of immune homeostasis are not well characterized.

**Methods:** In this study, by integrating the TCGA-LUAD database and Masked Somatic Mutation, we first conducted an investigation on the expression levels and mutation status of the KPNA family in patients with LUAD. Then, we constructed a prognostic model based on clinical features and the expression of the KPNA family. We performed functional enrichment analysis and constructed a regulatory network utilizing the differential genes in high-and low-risk groups. Lastly, we performed immune infiltration analysis using CIBERSORT.

**Results:** Analysis of TCGA datasets revealed differential expression of the KPNA family in LUAD. Kaplan-Meier survival analyses indicated that the high expression of *KPNA2* and *KPNA4* were predictive of inferior overall survival (OS). In addition, we constructed a prognostic model incorporating clinical factors and the expression level of *KPNA4* and *KPNA5*, which accurately predicted 1-year, 3-years, and 5-years survival outcomes. Patients in the high-risk group showed a poor prognosis. Functional enrichment analysis exhibited remarkable enrichment of transcriptional dysregulation in the high-risk group. On the other hand, gene set enrichment analysis (GSEA) displayed enrichment of cell cycle checkpoints as well as cell cycle mitotic in the high-risk group. Finally, analysis of immune infiltration revealed significant differences between the high-and low-risk groups. Further, the high-risk group was more prone to immune evasion while the inflammatory response was strongly associated with the low-risk group.

**Conclusions:** the KPNA family-based prognostic model reflects many biological aspects of LUAD and provides potential targets for precision therapy in LUAD.

## Introduction

Lung cancer is among the most prevalent tumors and contributes to about 21% of all cancer-related fatalities (Siegel et al., 2022). Non-small cell lung cancer (NSCLC) is the most common subtype of lung cancer that represents at least 85% of all cases of lung cancer. Histologically, NSCLC can be categorized into three types, namely, large cell carcinoma, lung squamous cell carcinoma (LUSC), and lung adenocarcinoma (LUAD), (Ko et al., 2018; Majem et al., 2020). Currently, the principal treatment modalities for lung cancer include targeted therapy, chemotherapy, radiotherapy, surgery, and immunotherapy (Catania et al., 2021). Due to the highly malignant nature of lung cancer, 5-year survival rates of patients with stage I to IIIA range from 14 to 49%, and those for stage IIIB to IV disease are <5% (Ko et al., 2018). LUAD is the most common subtype of lung cancer, accounting for approximately −40% of all cases (Yin et al., 2019). The 5-years overall survival (OS) rate of patients with LUAD is less than 20% (Wu et al., 2021). Therefore, exploration of the pathogenetic mechanism of LUAD and identification of potential therapeutic targets is a key research imperative.

Karyopherin alpha (KPNA) are nuclear transporters (NTRs) that consist of a cluster of basic amino acids, which selectively through the nuclear pore complex (NPC) (Hazawa et al., 2020; Miyamoto et al., 2020). NPC is composed of 30 nucleoporin (NUP) proteins, which is the sole channel between the nucleus and the cytoplasm (Hazawa et al., 2020). Active transport of proteins from the cytoplasm to the nucleus through NPC usually requires a carrier molecule that identifies the transport signal on the cargo, which is called nuclear localization signal (NLS) (Miyamoto et al., 2016). The classical mechanism of the passage of proteins into the nucleus is as follows: cargoes usually possess NLS that is initially detected by KPNA and then exhibits interaction with karyopherin b1 (KPNB1), and the created trimeric complex diffuses into the nucleus through NPC (Myat et al., 2018). The main role of KPNA in nucleocytoplasmic transport is to function as adaptor molecules that carry protein cargoes carrying NLS and Karyopherin beta (KPNB) from the cytoplasm to the nucleus (Miyamoto et al., 2016). In addition to its function in mediating nucleocytoplasmic transport, KPNA also has non-transport functions such as lamin polymerization, nuclear membrane formation, spindle assembly, protein degradation, cytoplasmic retention, cell surface function, gene expression, and mRNA-related function (Miyamoto et al., 2016). In addition, KPNA is increasingly recognized to have a central in cancer growth and progression (Wang et al., 2012; Xu et al., 2021).

The human type the KPNA family consists of seven subtypes, KPNA1, KPNA2, KPNA3, KPNA4, KPNA5, KPNA6, and KPNA7 (Miyamoto et al., 2016), and these subtypes exhibit 42–86% homology to one another (Oostdyk et al., 2019). The KPNA family can be further divided into three subfamilies based on sequence homology: α1, α2, and α3. The α1 subfamily comprises three members, KPNA1, KPNA5, and KPNA6. α2 subfamily comprises two members, KPNA2 and KPNA7. α3 subfamily comprises two members, KPNA3 and KPNA4 (Miyamoto et al., 2016; Myat et al., 2018). KPNA1 was the founding member of the α1 subfamily. The α2 and α3 subfamilies are known to have evolved through duplication of the founding KPNA, and to have developed cell and tissue-specific roles which facilitate development and differentiation in higher eukaryotes (Oostdyk et al., 2019). Aberrant expression of the KPNA family has been detected in multiple cancers, which was related to poor prognosis. For example, a study identified high KPNA1 expression in breast cancer, which was associated with poor overall survival (OS) (Tsoi et al., 2021). High KPNA2 expression in melanoma was linked to poor OS and disease-free survival (DFS) (Yang et al., 2020). High expression of KPNA2 has been identified in ovarian carcinoma and cervical cancer, which was associated with poor prognosis (Cui et al., 2021; Wang et al., 2021). High KPNA4 expression in liver cancer was shown to be associated with poor OS in patients (Xu et al., 2021).

The KPNA family plays varied roles in different types of malignancies. For example, KPNA1 was shown to modulate the nuclear import of NCOR2 splicing variant BQ323636.1 and thus promote tamoxifen resistance in breast cancer (Tsoi et al., 2021). The expression of KPNA2 in ovarian carcinoma can promote epithelial-mesenchymal transition (EMT), migration, and invasion. The expression of KPNA2 in colorectal cancer tissue was correlated with stage, differentiation status, and metastasis. Overexpression of KPNA2 indicated a poor prognosis in patients (Han and Wang, 2020). KPNA3 was shown to confer sorafenib resistance *via* TWIST-regulated EMT in advanced liver cancer (Hu et al., 2019). The expression of KPNA4 in prostate cancer was shown to promote metastasis through miR-708-KPNA4-TNF axes (Yang et al., 2017), and KPNA4 was found to enhance cancer cell proliferation and cisplatin resistance in cutaneous squamous cell carcinoma (Zhang et al., 2019). KPNA5, KPNA6, and KPNA1 binding regions can promote the proliferation of breast cancer cells (Kim et al., 2015). KPNA7 promotes cell growth and anchorage-independent growth, and reduces autophagy of pancreatic cancer cells (Laurila et al., 2014). Previous studies have reported overexpression of KPNA4 in LUAD and identified it as a potential key driver of the malignant phenotype (Hu et al., 2020). Nonetheless, the functional role and underlying mechanism of the KPNA family in LUAD are poorly understood.

In this study, we used the TCGA-LUAD database and Masked Somatic Mutation to evaluate the expression, mutation status, and prognostic value of the KPNA family in LUAD. We built a prognostic model for individuals on the basis of the clinical features and the expression of the KPNA family and analyzed the differences in mutational signature in the two risk groups. Next, we did a differential expression analysis, Kyoto Encyclopedia of Genes and Genomes (KEGG) pathway enrichment analysis, and Gene Ontology (GO) enrichment analysis in the two risk groups. Finally, we performed the analysis of immune infiltration in these groups. This is the first investigation to examine the function of the KPNA family in LUAD, as per our best knowledge. Our findings may avail both potential biomarkers and therapeutic targets against LUAD.

## Materials and methods

### Data acquisition and pretreatment

TCGA-LUAD expression profile data were acquired from UCSC Xena (http://xena.ucsc.edu/); the downloaded data type was count, and the count values were transformed to transcript per million (TPM) values in advance. Transcriptomic data from 594 patients in TCGA-LUAD, 535 tumor samples, and 59 normal samples were included in the current analysis. In addition, we selected “Masked Somatic Mutation” data as the somatic mutation data (*n* = 561) of LUAD patients from TCGA GDC (https://portal.gdc.cancer.gov/), processed these data using VarScan, and performed an analysis of somatic mutation using the maftools R package (Mayakonda et al., 2018). The copy number information (*n* = 531) of patients in TCGA-LUAD was downloaded in UCSC Xena, which assessed gene copy number variation (CNV).

In this analysis, we used the clinical information of 594 patients from TCGA-LUAD, including age, sex, survival status, and TNM stage. We matched patient IDs in the clinical database with the transcriptomic data as well as somatic mutation data above and removed samples with unavailable transcriptomic data and somatic mutation data.

The KPNA family (*KPNA1, KPNA2, KPNA3, KPNA4, KPNA5, KPNA6,* and *KPNA7*) expression profiles, mutation data, and CNV data were extracted *via* R languages for subsequent analysis.

### Differential expression analyses

Based on information in the TCGA-LUAD datasets, we divided the samples into tumor samples and normal samples and screened out differentially expressed genes (DEGs) utilizing the DESeq2 package. The screening criteria were log2 (fold change) > 1.0 and *p*-value < 0.05 (Love et al., 2014). Subsequently, differential expression analysis was performed using the DESeq2 package to determine the expression profiles of low-and high-risk groups. The screening criteria were log2 (fold change) > 2.0 and adj. *p*-value < 0.05. Volcano plots were plotted using package ggplot2, heat maps were drawn using package pheatmap to demonstrate the differential gene expression.

### Establishment of the prognostic model

Kaplan-Meier method in conjunction with the log-rank test was utilized for survival analysis to establish the link between high/low expression of the KPNA family genes and OS.

To determine the predictive power of the KPNA family for the prognosis of LUAD individuals, we performed univariate Cox regression analysis, LASSO regression analysis, and multivariate Cox regression analysis based on the TCGA-LUAD to identify independent prognostic factors, and created a prognostic model. First, univariate Cox proportional regression analysis was utilized to investigate the link between the expression levels of genes in the KPNA family and OS; genes with an adjusted *p-*value < 0.1 were retained. Subsequently, to eliminate the effect of multicollinearity, we used the LASSO algorithm to screen meaningful variables in univariate Cox regression analysis. Then we performed a stepwise regression analysis using multivariate Cox regression to discover independent prognostic factors. Finally, optimized gene expression and correlation estimated Cox regression coefficients were taken into consideration to generate a risk score formula: risk score = (exp-Gene1*coef-Gene1) + (exp-Gene2*coef-Gene2)+……+(exp-Gene*coef-Gene).

The participants were then classified into the aforementioned two risk groups as per the given risk score. Kaplan-Meier analysis and log-rank test were performed to compare OS in the two groups applying the survival package. Additionally, receiver operating characteristic (ROC) curve analysis evaluated the survival predictive value of the risk score. The area under ROC curves (AUC) values were derived utilizing the R package timeROC.

After detection of independent prognostic factors, we combined clinical information such as age, sex, stage, and other factors to establish a nomogram for prognostic assessment of LUAD patients. In particular, we evaluated the prognostic outcomes at 1, 3, and 5 years, correspondingly. The reliability of the model was assessed by plotting the calibration curve.

### Construct functional enrichment analysis and regulatory network

We did GO enrichment analysis as well as KEGG pathway enrichment analysis of the differentially expressed genes of two risk groups utilizing the clusterProfiler R package and R package GOPlot (Ogata et al., 1999; Ashburner et al., 2000; Yu et al., 2012). GSEA was instrumental in developing the gene expression matrix with clusterProfiler R package; “c2. cp.all.v7.0. symbols” was chosen as a reference gene set. In addition, false discovery rate (FDR) < 0.25 with *p* < 0.05 denotes substantial enrichment (Suarez-Farinas et al., 2010). Based on the “c2. cp.all.v7.0. symbols” gene set, we utilized the R package Gene set variation analysis (GSVA) on the basis of the gene expression matrix for each sample, calculated the related pathway scores, and generated the Heat maps using the ssGSEA method (Hänzelmann et al., 2013).

Using the STRING protein-protein interactions database, we evaluated the link between the hub genes and their interactions and exported the results; core genes were thoroughly screened with the CytoHubba Plugin in Cytoscape (Chin et al., 2014).

In addition, hub genes-miRNA regulation analysis and transcription factors-target genes regulatory network analysis were performed with NetworkAnalyst (http://www.networkanalyst.ca/NetworkAnalyst). Results were finally exported from Networkanalyst, and miRNA-hub genes and transcription factors-hub genes regulatory network plotted using Cytoscape software.

### Analysis of immune cell infiltration

We performed deconvolution with transcriptome matrix using the CIBERSORT algorithm (which is premised on the linear support vector regression principle) and assessed the cellular composition and the abundance of immune cells in the mixed infiltrate (Newman et al., 2015). Gene expression matrices data were uploaded onto the CIBERSORT, and after filtering the outputs (*p-*value < 0.05), we obtained the matrix of infiltrating immune cells. Bar graphs were plotted using R package ggplot2 to demonstrate the distributions of 22 types of infiltrating immune cells in every sample. In addition, we studied the correlation of two risk groups with immune and inflammation by extracting HLA family-related genes (MHC class I and II) and complement-related genes.

### Statistical analysis

The R software (version 4.0.2) performed all the analyses and data processing. Between-group variations with respect to normally distributed continuous variables were investigated with the aid of the Student’s *t-*test, whereas those with respect to non-normally distributed variables were investigated utilizing the Mann-Whitney U test (Wilcoxon’s rank-sum test). Additionally, for between-group differences with respect to categorical variables, the Chi-squared test or Fisher exact test was used. Correlation between different genes was assessed using Spearman correlation analysis. Kaplan-Meier survival analyses were done through the utilization of the R package survival and the between-group differences in survival outcomes were assessed using the log‐rank test. Univariate as well as multivariate Cox regression analyses were utilized to ascertain the independent prognostic factors. Two-sided *p* values < 0.05 denoted statistical significance for all analyses.

## Results

### Aberration of the KPNA family in TCGA-LUAD

**FIGURE 1:**
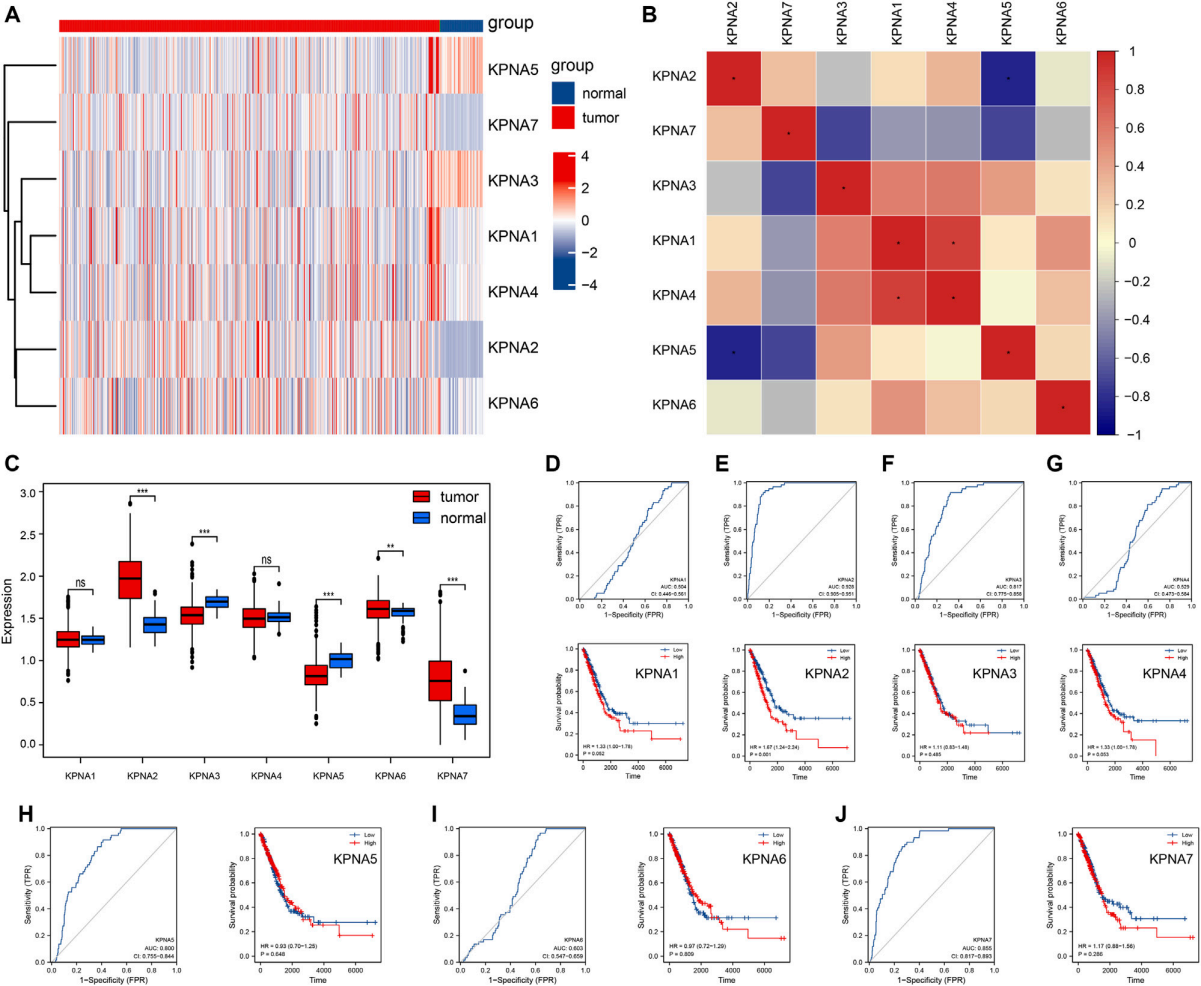
Expression patterns of the KPNA family in TCGA-LUAD **(A)** Heat maps of gene expressions of the KPNA family **(B)** Heat map of gene-gene correlations in the KPNA family **(C)** Boxplots of the KPNA family genes between the normal and tumor tissues **(D–J)** ROC curve showing group differences and the Kapla-Meier curves showing survival differences. * represents *p* < 0.05; ** represents *p* < 0.01; *** represents *p* < 0.001; ns represents no significant difference (*p* > 0.05).

First, we extracted the KPNA family from the TCGA-LUAD datasets, which included *KPNA1, KPNA2, KPNA3, KPNA4, KPNA5, KPNA6,* and *KPNA7*, and the details are shown in Supplementary Table S1. We plotted the heatmaps of the KPNA family and found a non-uniform trend in their expression with no significant correlations between them (Figures 1A,B). We identified differential expression of *KPNA2, KPNA3, KPNA5, KPNA6,* and *KPNA7*. Compared with normal tissue, *KPNA2, KPNA6,* and *KPNA7* were highly expressed in LUAD, while *KPNA3* and *KPNA5* expression were decreased in LUAD (Figure 1C). Subsequently, we plotted ROC curves, which clearly showed the discriminative value of these genes in differentiating between tumor samples and non-tumor samples. The AUC values of *KPNA2, KPNA3, KPNA5,* and *KPNA7* were >0.7, which indicated a promising discriminating ability. In addition, we did Kaplan-Meier survival analysis to identify genes that affect the prognosis in LUAD. The expression of *KPNA2* and *KPNA4* was found to affect the OS of LUAD individuals, and the patients with high expression of *KPNA2* and *KPNA4* showed a much worse prognosis (Figures 1D–J).

**FIGURE 2:**
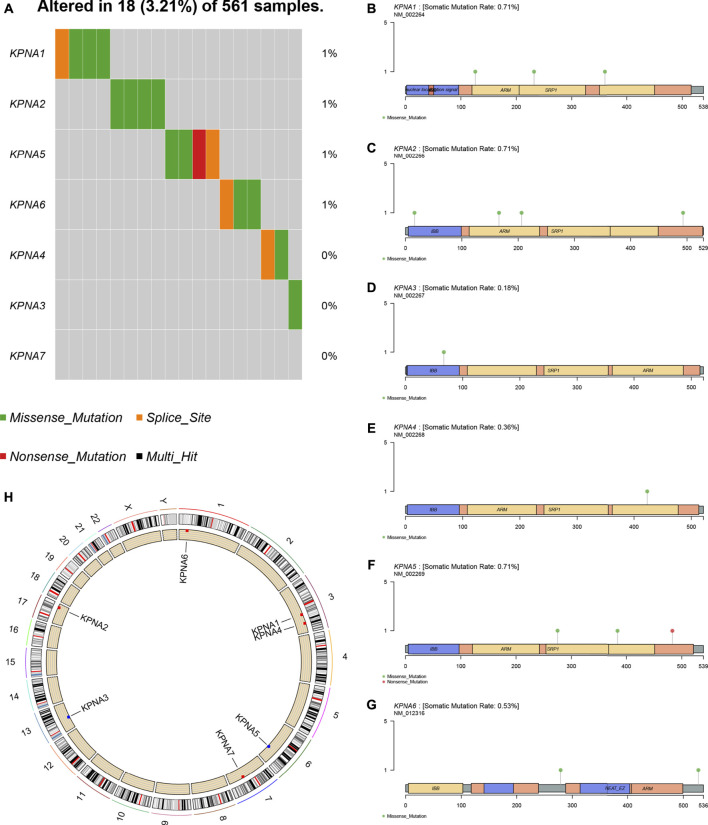
Mutations and copy number variations (CNV) of the KPNA family **(A)** Mutation frequency of the KPNA family **(B–G)** Mutated sites of the KPNA family **(H)** Copy number alterations of the KPNA family; red dots indicate that amplifications are greater than deletions while blue dots indicate that deletions are greater than amplifications.

The panorama of gene mutations was displayed in TCGA-LUAD datasets; missense mutations accounted for the majority of mutations, single-nucleotide polymorphisms (SNPs) occurred more frequently than deletions or insertions, and C>A was most frequently identified in single nucleotide variants (SNVs) among patients with LUAD (Supplementary Figures S1A,B). Subsequently, we extracted the KPNA family information and analyzed the mutational signatures. The frequency of overall the KPNA family mutations was low, and the mutation types were primarily missense mutations (Figure 2A). We plotted the lollipop diagrams according to mutational signatures (Figures 2B–G). In addition, we analyzed CNV changes according to the information on the CNV of the KPNA family. As shown in Figure 2H, the copy number amplifications of *KPNA1, KPNA2, KPNA4, KPNA6,* and *KPNA7* in total samples were higher than the copy number deletions, but the copy number amplifications of *KPNA3* and *KPNA5* were lower than the copy number deletions.

### Creation of prognostic model based on the KPNA family

**FIGURE 3:**
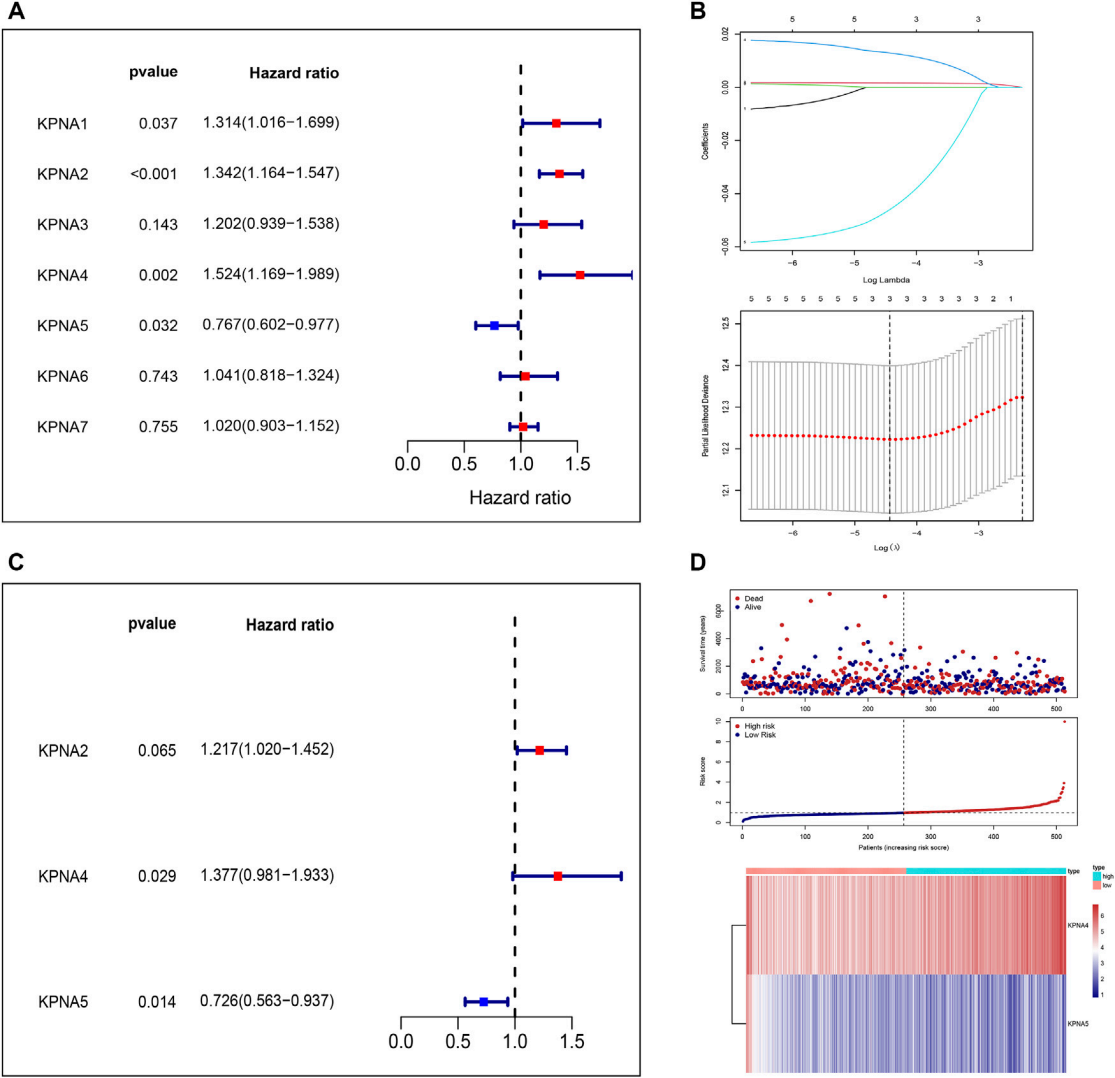
Independent prognostic factors of the KPNA family **(A)** Forest plot of univariate Cox regression analysis of the KPNA family **(B)** Lasso regression model of the KPNA family **(C)** Forest plot of multivariate Cox regression analysis of the KPNA family **(D)** Calculated risk score and the heat maps of risk factors based on the findings of multivariate Cox regression analysis.

**FIGURE 4:**
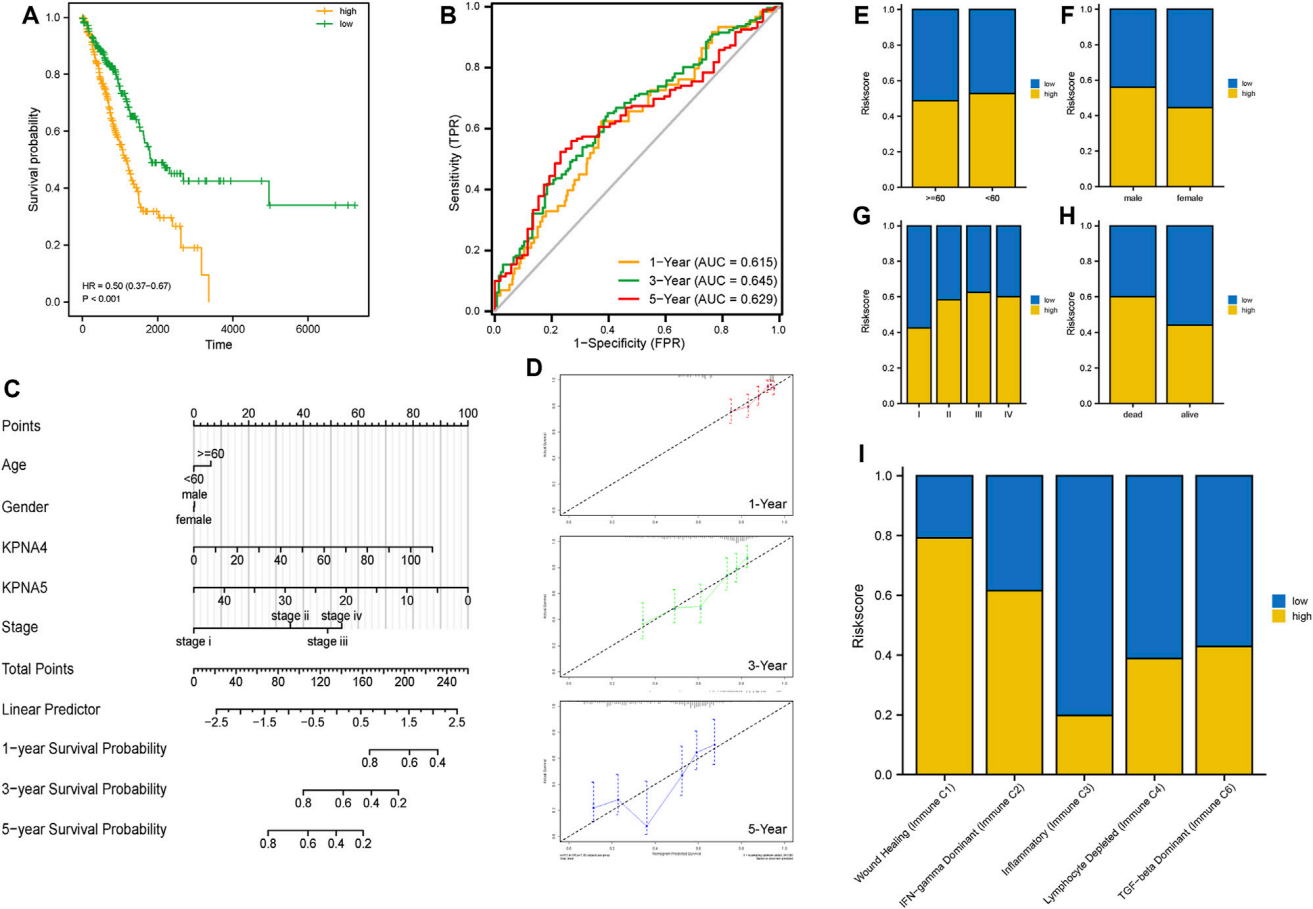
Evaluation of prognostic model and correlation analysis of clinical features **(A)** Analysis of prognosis in the two risk groups **(B)** ROC curves displaying the predictive value of the models for 1-year, 3-year, and 5-year survival outcomes **(C,D)** Prognostic nomogram and calibration curves according to clinical factors and the expression of KPNA4 and KPNA5 **(E–I)** Differences in clinical features between the two risk groups.

We conducted a univariate Cox regression analysis to detect the KPNA family genes linked to the prognosis of LUAD patients. Four genes were discovered to be linked to survival. To further screen the genes associated with prognosis, we screened the genes using LASSO regression analysis and Cox regression analysis and eventually identified *KPNA4* and *KPNA5* as independent prognostic factors (Figures 3A–C). As per their expression values and regression coefficients, we derived the risk score for LUAD specimens and plotted the heatmaps to visualize the distribution of samples in the two risk groups (Figure 3D). We conducted a survival analysis of LUAD individuals utilizing their risk score-based grouping; the findings affirmed that patients in the high-risk group experienced a poor prognosis (Figure 4A). ROC curve analysis indicated good predictive efficacy of risk score-based grouping for 1-year, 3-years, and 5-years survival outcomes (1-year AUC = 0.615, 3-years AUC = 0.645, 5-years AUC = 0.629) (Figure 4B).

Subsequently, we constructed a nomogram incorporating age, sex, clinical stage, and the expression level of *KPNA4* and *KPNA5* for prognostic assessment of LUAD patients (Figure 4C). Through calibration curves, we found that the prognostic model for 1-year, 3-years, and 5-years had high reliability (Figure 4D). Additionally, we performed risk stratification based on different factors including age, sex, clinical stage, survival status, and immune subtypes. The results affirmed that there were no remarkable differences between the two risk groups with respect to age or sex; however, there were substantial differences between the two risk groups in terms of clinical stage and immune subtypes (Figures 4E–I).

### Comparison of tumor mutation burden and microsatellite instability utilizing risk score

**FIGURE 5:**
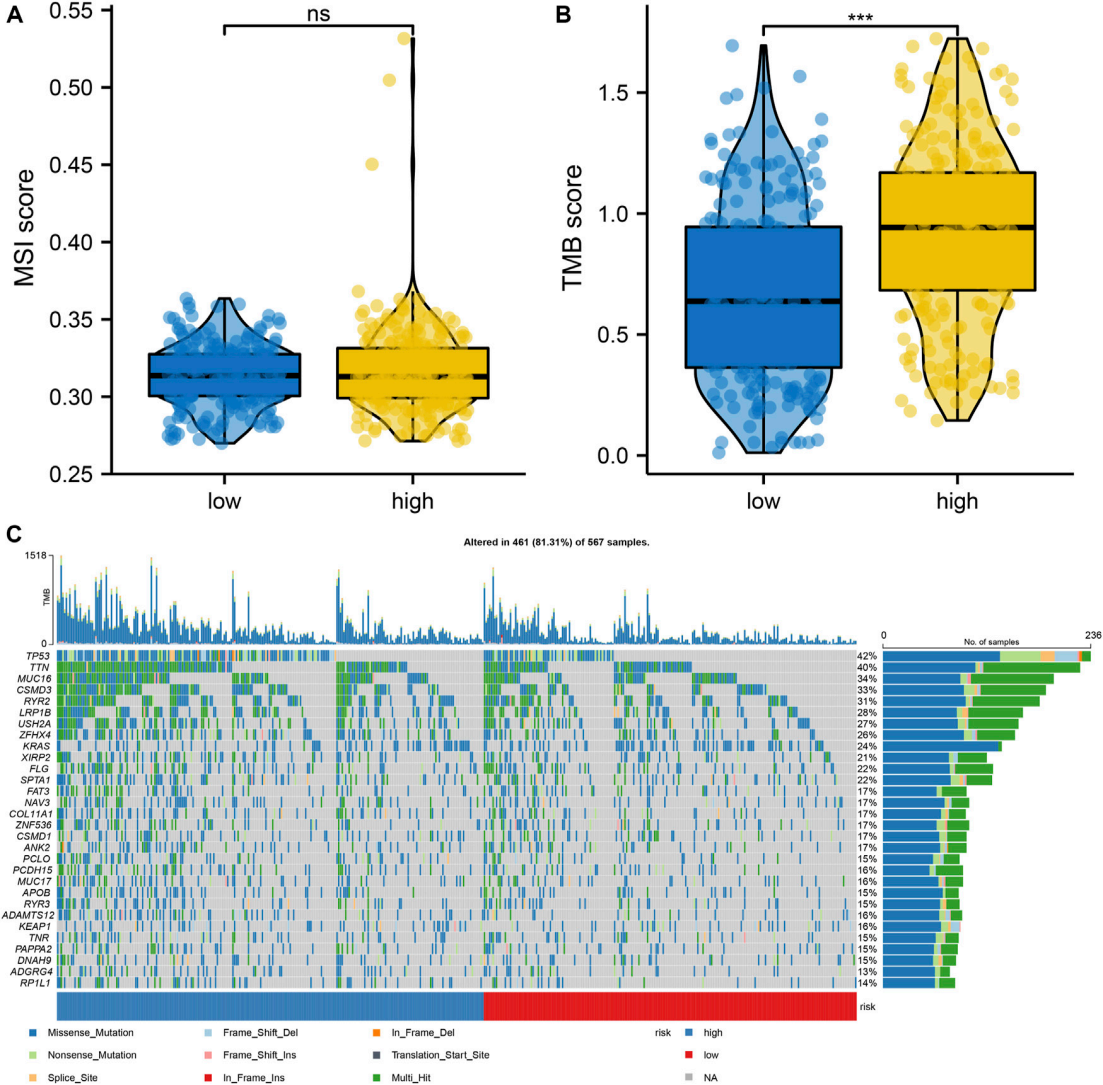
Differences in mutation signatures between high-and low-risk groups **(A)** Differences in MSI score between high- and low-risk groups **(B)** Differences in TMB score between high-and low-risk groups **(C)** Differences in the top 30 mutant genes between high-and low-risk groups. *represents *p* < 0.05; ** represents *p* < 0.01; *** represents *p* < 0.001; ns represents no significant difference (*p* > 0.05).

We further compared the mutational signatures between the two groups utilizing the risk score. There were no remarkable differences in MSI scores between the two risk groups, but the high-risk group had greater TMB scores in contrast to the low-risk group (Figures 5A,B). Subsequently, we analyzed the top 30 mutant genes of the two risk groups and ascertained variations in genetic mutations between them (Figure 5C).

### Differential expression analysis and functional enrichment analysis of high-and low-risk groups

**FIGURE 6:**
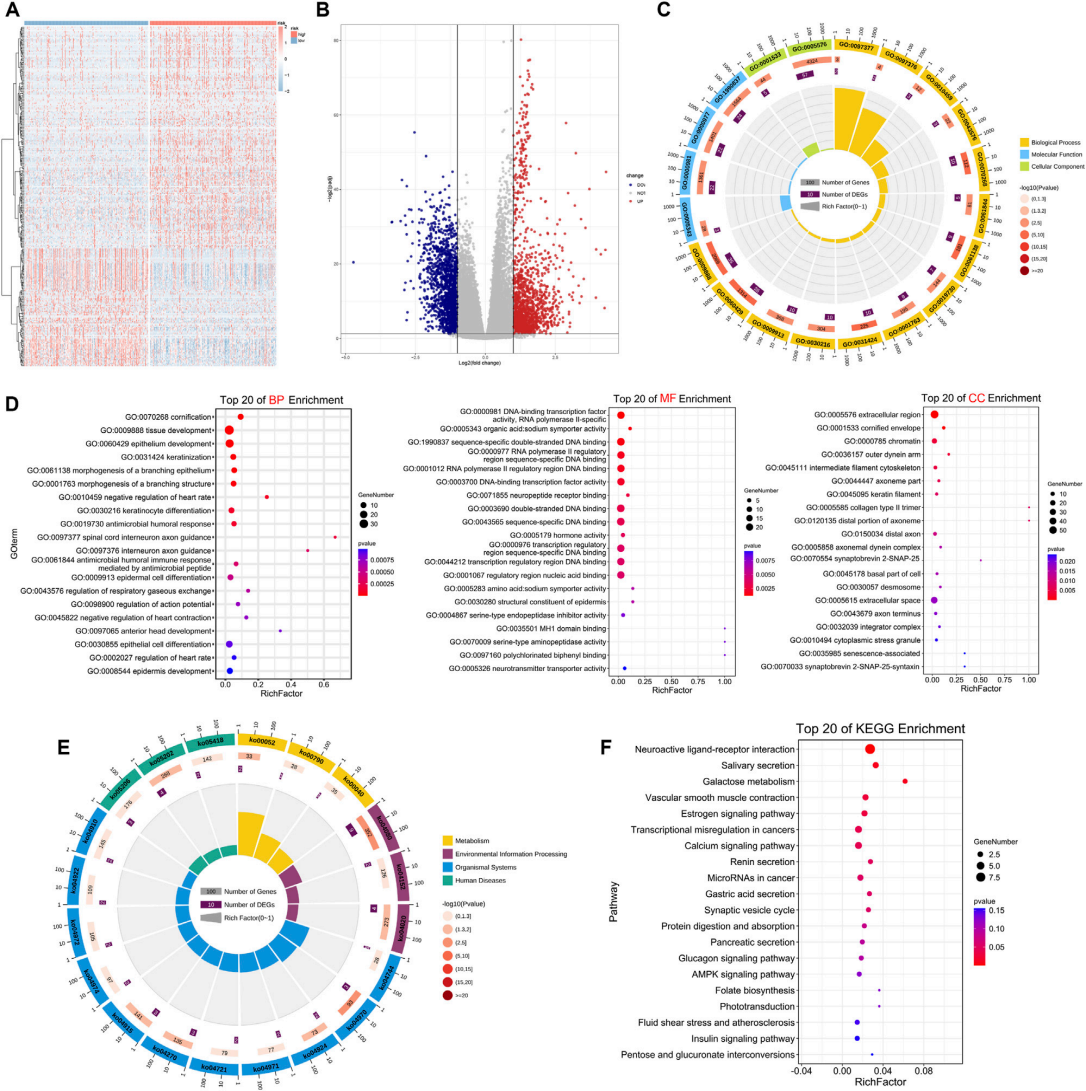
Differential expression analysis and functional enrichment analysis of the two risk groups **(A,B)** Heat map and volcano plot of differential expression in high-and low-risk groups **(C)** GO analysis of the two risk groups. Outer circle, GO term; cylindrical of inner circle, number of enriched genes; yellow, BP (Biological Process); blue, MF (Molecular Function); green, CC (Cellular Component) **(D)** Top 20 of BP, MF, CC **(E)** KEGG analysis of high-and low-risk groups. Outer circle, the number of KEGG pathways; inner circle, number of enriched genes; yellow, metabolism; blue, organismal systems; green, human diseases; purple, environmental information processing **(F)** Top 20 of KEGG pathways enrichment.

According to the low-and high-risk groups, we did a differential analysis of all genes within the expression profiles in the TCGA cohort using the volcano plots and heat maps (Figures 6A,B). Pathway enrichment analysis, as well as GO enrichment analysis, were performed on DEGs separately (Supplementary Tables S2, S3). GO enrichment analysis included molecular function (MF), biological process (BP), and cellular component (CC). The key DEGs enriched the following principal biological processes: epithelium development, cornification, tissue development, and morphogenesis of a branching epithelium, morphogenesis of a branching structure; the principal aggregation of cellular components was as follows: extracellular region, cornified envelope, and chromatin. The principal enriched molecular functions were as follows: DNA-binding transcription factor activity, sequence-specific double-stranded DNA binding, and amino acid sodium symporter activity (Figures 6C,D). The pathway enrichment was mainly enriched in Neuroactive ligand-receptor interaction, Salivary secretion, Galactose metabolism, Vascular smooth muscle contraction, and Transcriptional dysregulation in cancers (Figures 6E,F).

Subsequently, we constructed PPI networks by STRING databases to identify the hub genes and reveal their potential interactions. First of all, we built protein interaction networks by DEGs and the minimum score of interactions was set to 0.7 (Supplementary Figure S2A). We additionally determined the most relevant genes in the PPI networks by the Cytohubba plugin and 15 genes were regarded as hub genes: *SPANXD, MAGEA4, MAGEC1, SPANXC, CTAG2, MAGEA10, CT45A1, MAGEA1, MAGEA1, MAGEC2, SPRR2D, KRT6A, KRT14, CASP14*, and *SPRR2E* (Supplementary Figure S2B).

We also predicted the potential miRNAs which regulate the 15 hub genes by the Networkanalyst databases; the final subnetwork contained 49 nodes (i.e., miRNA) and 11 seeds (i.e., matched hub genes) (Supplementary Figure S2C). Similarly, we obtained the transcription factors-hub genes regulatory networks based on the JASPAR databases, the final contained 14 seeds (i.e., hub genes) and 46 nodes (i.e., transcription factors) (Supplementary Figure S2D).

**FIGURE 7:**
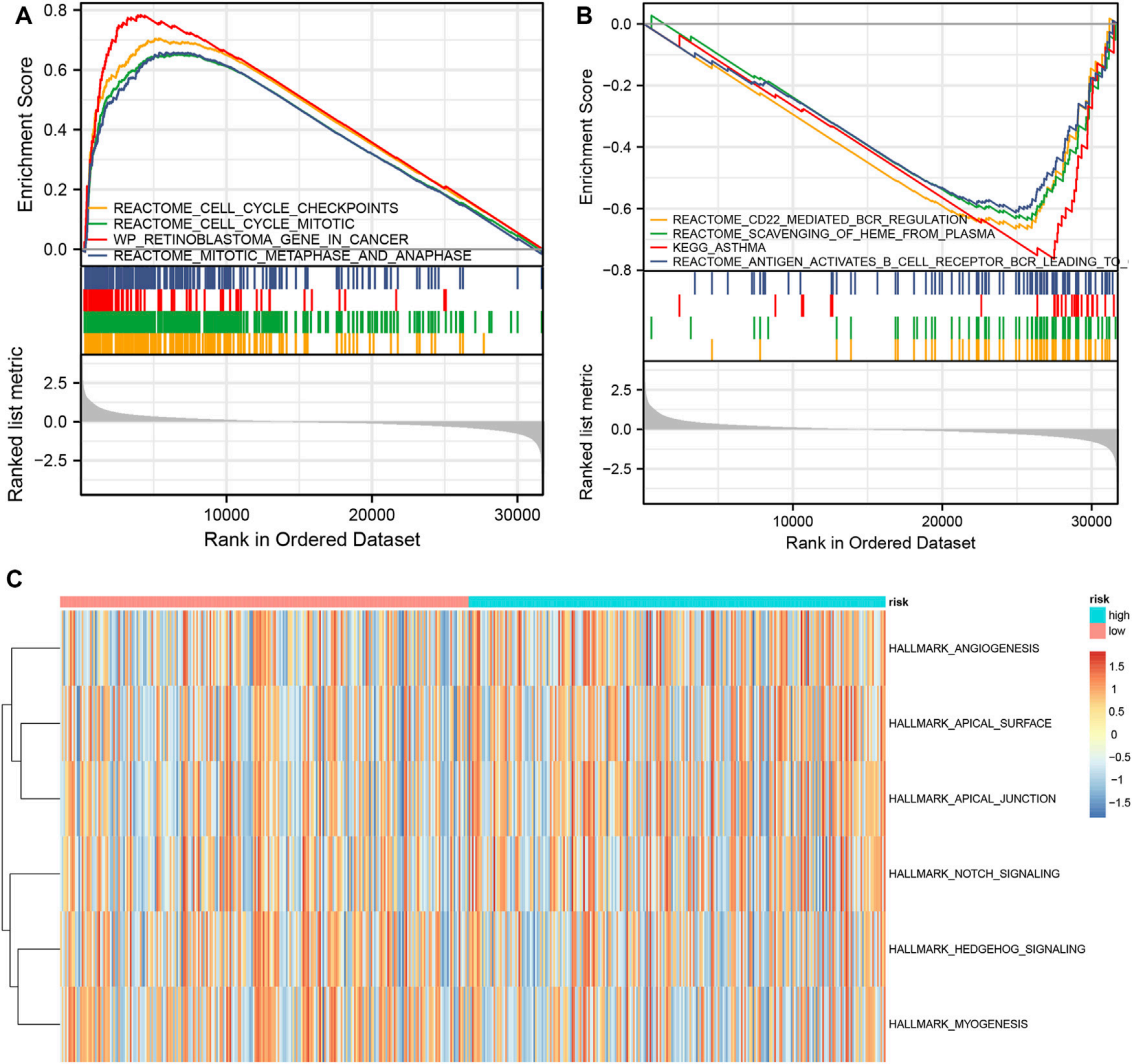
GSVA and GSEA analysis of high- and low-risk groups **(A)** Main enriched pathways in the high-risk group **(B)** Main enriched pathways in the low-risk group **(C)** Differential gene sets based on the hallmark gene sets.

Subsequently, we carried out GSEA between the two risk groups to identify remarkably enriched pathways (*p*-value < 0.05) (Supplementary Table S4). The GSEA results showed enrichment of cell cycle checkpoints, cell cycle mitotic, retinoblastoma gene in cancer, mitotic metaphase, and anaphase in the high-risk group. CD22 mediated BCR regulation, heme scavenging from plasma, asthma, and antigen activates B cell receptor BCR resulting in the generation of second messengers were enriched in the low-risk group (Figures 7A,B). GSVA findings ascertained that there were variations in a total of six gene sets between the two risk groups, according to the screening of the hallmark gene sets, for example, angiogenesis, apical surface, and apical junction (Figure 7C).

### Analysis of immune infiltration in the high-and low-risk groups

**FIGURE 8:**
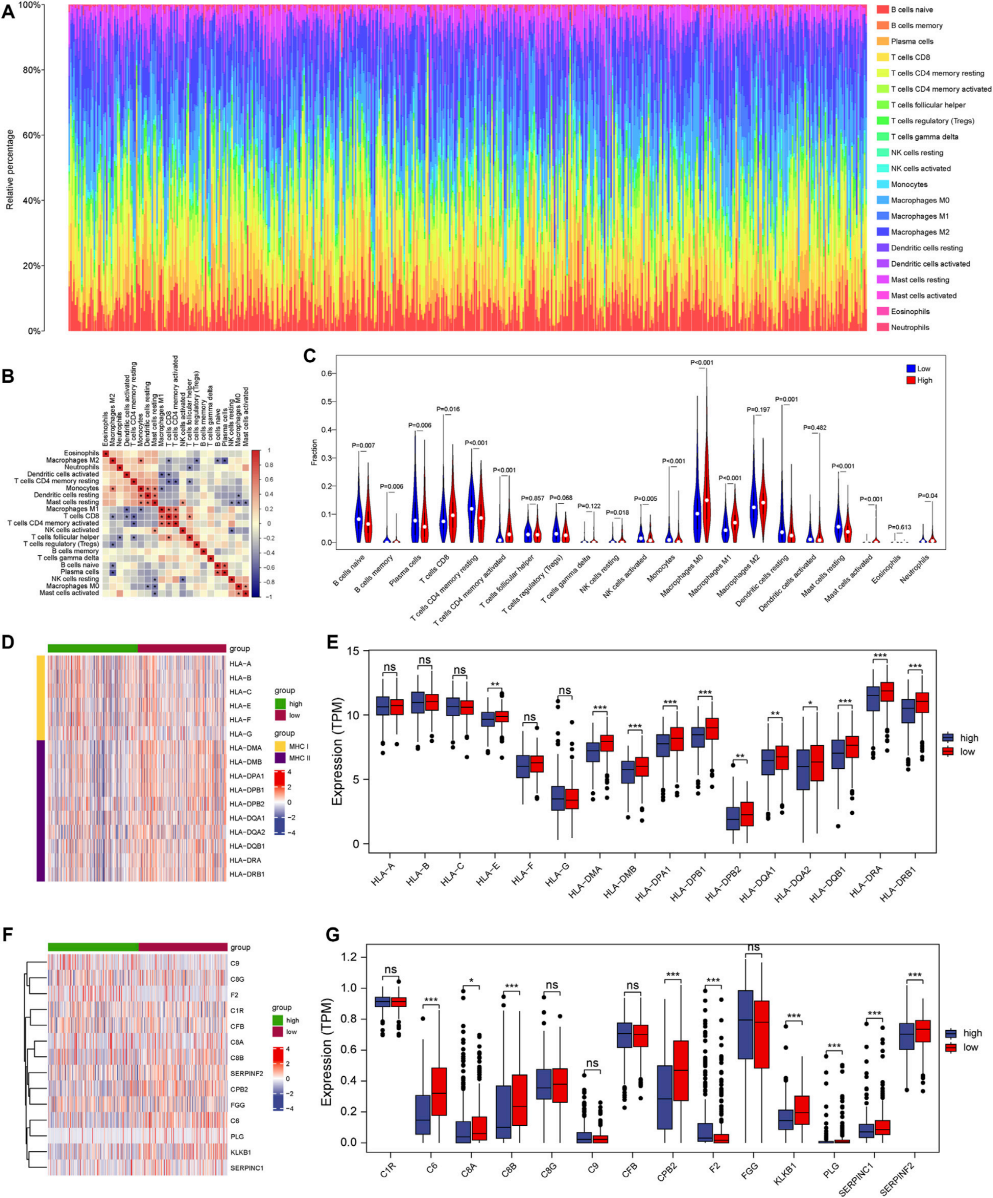
Immune infiltration in the two risk groups **(A)** The panorama of 22 immune cell infiltrates **(B)** Correlation analyses of 22 immune cell types **(C)** Differences in the immune cell infiltration between high-and low-risk groups **(D,E)** Differential expression of HLA gene family between the two risk groups **(F,G)** Differential expression of complement-related genes in the two risk groups. * represents *p* < 0.05; ** represents *p* < 0.01; *** represents *p* < 0.001; ns represents no significant difference (*p* > 0.05).

After ranking based on the risk score, the immune cell infiltration for each sample in the TCGA LUAD is shown in the bar graphs. The infiltration scores and correlation analysis between the 22 immune cells were obtained by the CIBERSORT algorithm, respectively (Figures 8A,B). We further evaluated the differences in immune cell infiltrates in the two risk groups. As shown in Figure 8C, the infiltration scores for naive B cells, plasma cells, CD4^+^ T cells memory resting T cells, and resting dendritic cells were lower in the high-risk group than in the low-risk group; however, the infiltration scores for CD8^+^ T cells and M0 Macrophages were greater in the high-risk group. We computed the correlation of the expression level of *KPNA4* and *KPNA5* and various types of immune cells by Spearman’s correlation analysis (Supplementary Figures S3, S4). Additionally, we combined the genes related to immunity and inflammation (for example, HLA family and complement-related genes), and analyzed the differences in the two risk groups. We found that the MHC-II family was decreased in the high-risk group, and the main function of the MHC-II gene is antigen-presenting. This suggested that the antigen-presenting function might be affected in the high-risk group (Figures 8D,E). Additionally, there were variations of complement-related genes in both groups, which illustrated a close association with inflammation (Figures 8F,G).

## Discussion

Due to its highly malignant nature and a paucity of methods for early diagnosis, LUAD is linked to high incidence as well as mortality rates. Therefore, recognition of particular principal molecular pathways and extensively sensitive, reliable biomarkers is required to improve the early diagnosis and survival outcomes of LUAD patients. Previous investigations have demonstrated the relationship of the KPNA family genes with tumor progression (Wang et al., 2012; Xu et al., 2021). However, there is a lack of in-depth characterization of the role of the KPNA family in LUAD. This is the first investigation to develop a prognostic model premised on the expression of the KPNA family genes, as per our best knowledge. Enrichment analysis revealed the involvement of the KPNA family in transcription, cell cycle, immune infiltration, and inflammatory response, which are tumor-related processes. Thus, our findings may be useful in the development of future investigations to determine patient prognosis and to recognize candidate therapeutic targets in LUAD individuals.

We explored the connection between the KPNA family expression and the OS of patients. High expression of *KPNA2* and *KPNA4* were predictive of inferior OS. *KPNA4* has previously been identified as a tumor promoter gene in some cancers (Wang et al., 2015). For example, high expression of *KPNA4* in cutaneous squamous cell carcinoma was discovered to enhance cancer cell proliferation as well as cisplatin resistance (Zhang et al., 2019). Inhibition of *KPNA4* attenuated prostate cancer metastasis (Yang et al., 2017). Regulating upstream modulators facilitates angiogenesis as well as progression in lung cancer by targeting the miR‐340‐5p/KPNA4 axis (Li et al., 2020). A previous study identified overexpression of *KPNA2* in NSCLC, and *KPNA2* was identified as a potential biomarker for NSCLC (Wang et al., 2011). These studies support our conclusions that *KPNA2* and *KPNA4* may be useful prognostic biomarkers for LUAD patients.

KEGG enrichment analysis showed transcriptional dysregulation in cancers enriched with DEGs in the high-risk group. Transcription factors serve as a group of sequence-specific binding proteins that can activate or suppress transcription through transactivation or transrepression domains. Transcription factors have been linked to the pathogenesis of a variety of human diseases (including cancers); these account for approximately 20% of all oncogenes identified so far (Lambert et al., 2018). Previous literature reports have displayed the involvement of transcription factors in regulating cell proliferation, differentiation, apoptosis, and their remarkable function in the onset and development of tumors (Sever and Brugge, 2015). Dysregulation of principal transcriptional modulators not only defines the cancer phenotype but is important for its development (Gonda and Ramsay, 2015). Our results suggest that the KPNA family may influence the transcriptional dysregulation in LUAD. Therefore, it is important to study the mechanism of transcriptional dysregulation of the KPNA family in LUAD.

In this study, we found that cell cycle checkpoints and cell cycle mitotic were enriched in the high-risk group. Cell cycle checkpoints are biochemical signaling mechanisms that detect DNA damage or chromosomal dysfunction and trigger a series of sophisticated cellular repair responses (Wu et al., 2005). Typically, cell cycle checkpoints are disrupted in most malignancies and serve a vital function in maintaining genomic integrity and inactivating checkpoint genes (Zheng et al., 2010). In previous research, impaired function of cell cycle checkpoints was found to raise the risk of lung cancer (Wu et al., 2005). Mitosis is the critical stage of the cell cycle, involving the passage of one of the sister chromatids to each of the daughter cells. Therefore, precise regulation of mitosis is essential for the maintenance of chromosome stability in human cells (Pines, 2006). Aberrant mitotic progression leads to chromosomal missegregation, contributing to carcinogenesis (Kops et al., 2005; Holland and Cleveland, 2009; Schvartzman et al., 2010). Our study identified significant enrichment of these two pathways in the high-risk group, which additionally validated the accuracy of the risk prediction model constructed in this study.

The tumor microenvironment (TME) is a heterogeneous system consisting of immune cells, cancer cells, and an extracellular matrix (Hoadley et al., 2014; Warrick et al., 2019). The roles for immune homeostasis similar to a buffering system. While the immune system is constantly stimulated and dampened, the system is maintained at a relatively stable steady state (da Gama Duarte et al., 2018). In this study, the infiltration scores for naive B cells, plasma cells, CD4^+^ T cells memory resting T cells, and resting dendritic cells were lowered in the high-risk groups than in the low-risk groups, but the infiltration scores for CD8^+^ T cells, M0 Macrophages were elevated in the high-risk group. This could lead to different responses to immunotherapies in the two risk groups. The purpose of immunotherapy is to alter the environment, and thereby, the equilibrium of the response. Therefore, the sensitivity of immunotherapy in the two risk groups also remains unexplored.

Immune evasion is a significant feature of cancer, and inhibition of HLA gene levels may lead to attenuated antigen presentation, facilitating immune evasion (McGranahan et al., 2017). HLA family genes were decreased in the high-risk group, which suggests that the high-risk group was more prone to immune evasion and thus have a worse prognosis. These results are consistent with our survival analysis. Additionally, we studied the expression of inflammation-related genes in the two risk groups and captured the down-regulation of complement-related genes in the high-risk group. These findings suggest that inflammation was strongly associated with the low-risk group.

This is the first-ever report on the association of the KPNA family expression with survival outcomes of patients with LUAD. Therefore, the KPNA family may potentially serve as a novel prognostic biomarker in patients with LUAD and provide novel targets for LUAD immunotherapy. However, this was bioinformatics research and most of the findings were generated from public databases and bioinformatics analysis. Further *in vitro* and *in vivo* experiments are required to validate our findings.

In conclusion, we found that *KPNA2* and *KPNA4* are potential prognostic markers. We created a prognostic model on the basis of the expression level of the KPNA family, which was shown to accurately predict prognosis. This prognostic model reflects many aspects of LUAD biology and provides new insights into precision therapy for LUAD. In the future, a lot of basic experiments need to be carried out to validate the applicability and accuracy of this model.

## Data Availability

The original contributions presented in the study are included in the article/Supplementary Material, further inquiries can be directed to the corresponding authors.
