# Supplementary material for: Comprehensive analysis of karyopherin alpha family expression in lung adenocarcinoma: Association with prognostic value and immune homeostasis
Source: Front Genet. 2022 Aug 3;13:956314. doi: 10.3389/fgene.2022.956314 (PMC9382304; doi:10.3389/fgene.2022.956314)
Supplement: Supplementary file 1 [file DataSheet1.docx]

Supplementary Material

# Supplementary Figures and Tables

## Supplementary Figures


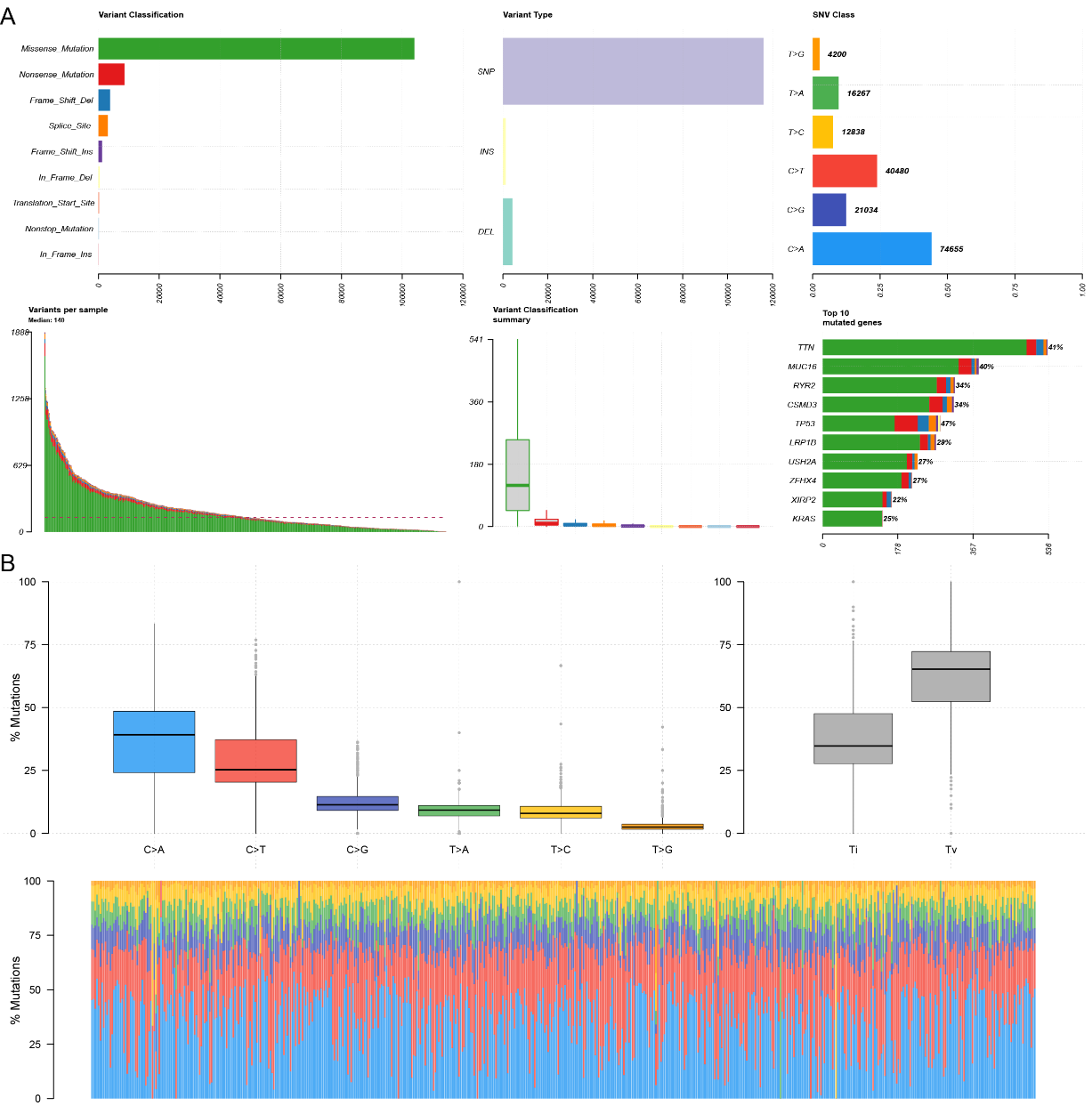


**Supplementary Figure 1.** Panorama of mutations in TCGA-LUAD. **(A-B)** The majority are missense mutations; single-nucleotide polymorphisms (SNPs) occurred at significantly higher frequencies than deletions and insertions, C>A is the most common single-nucleotide variant (SNV).


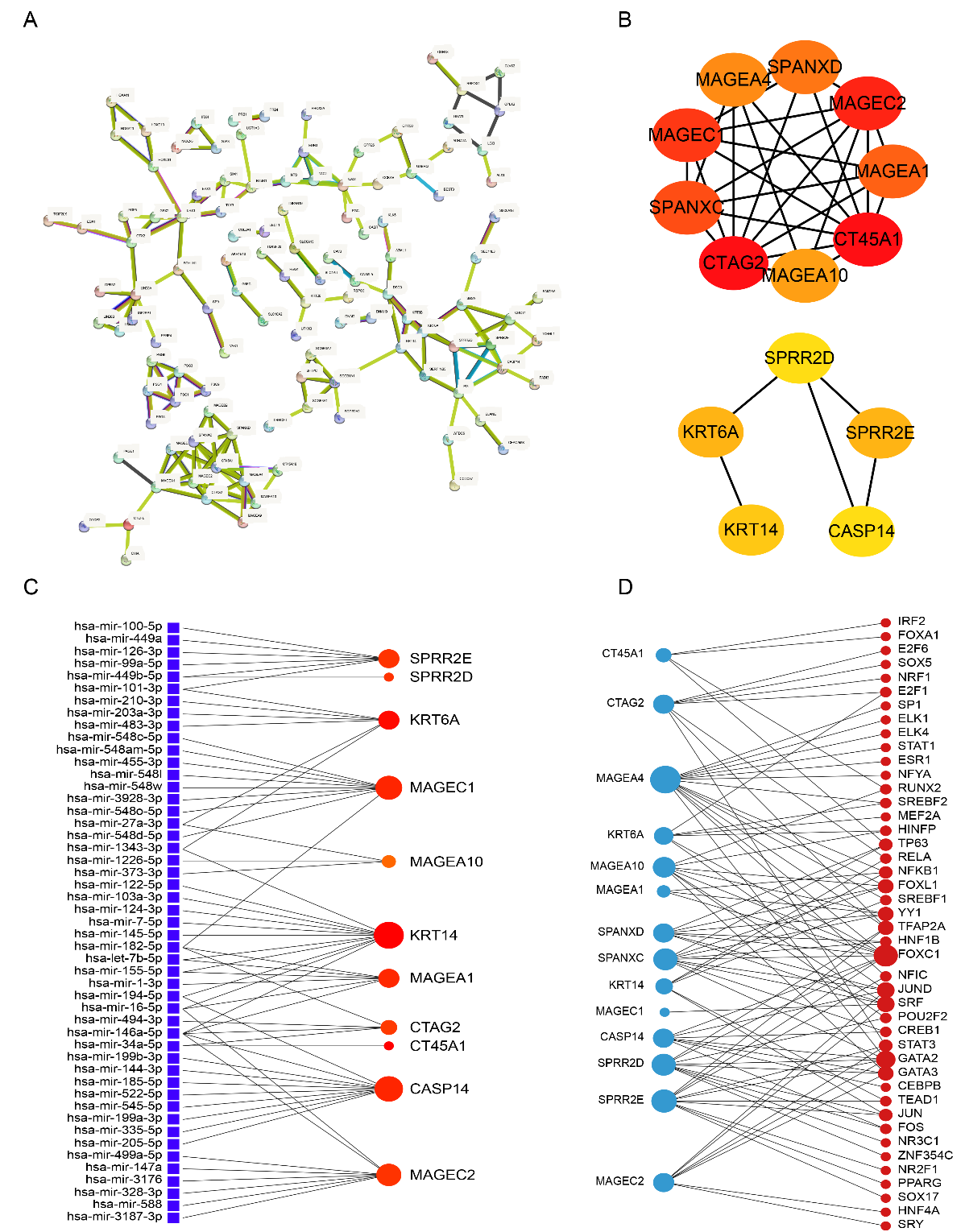


**Supplementary Figure 2.** Constructed regulatory network. **(A)** Protein–protein interaction network of differentially-expressed genes (DEGs) from the STRING database. **(B)** 15 hub genes were identified through CytoHubba. **(C)** 49 miRNA and 11 hub genes were identified through Networkanalyst. **(D)** 14 hub genes and 46 transcription factors were identified through Jaspar database.


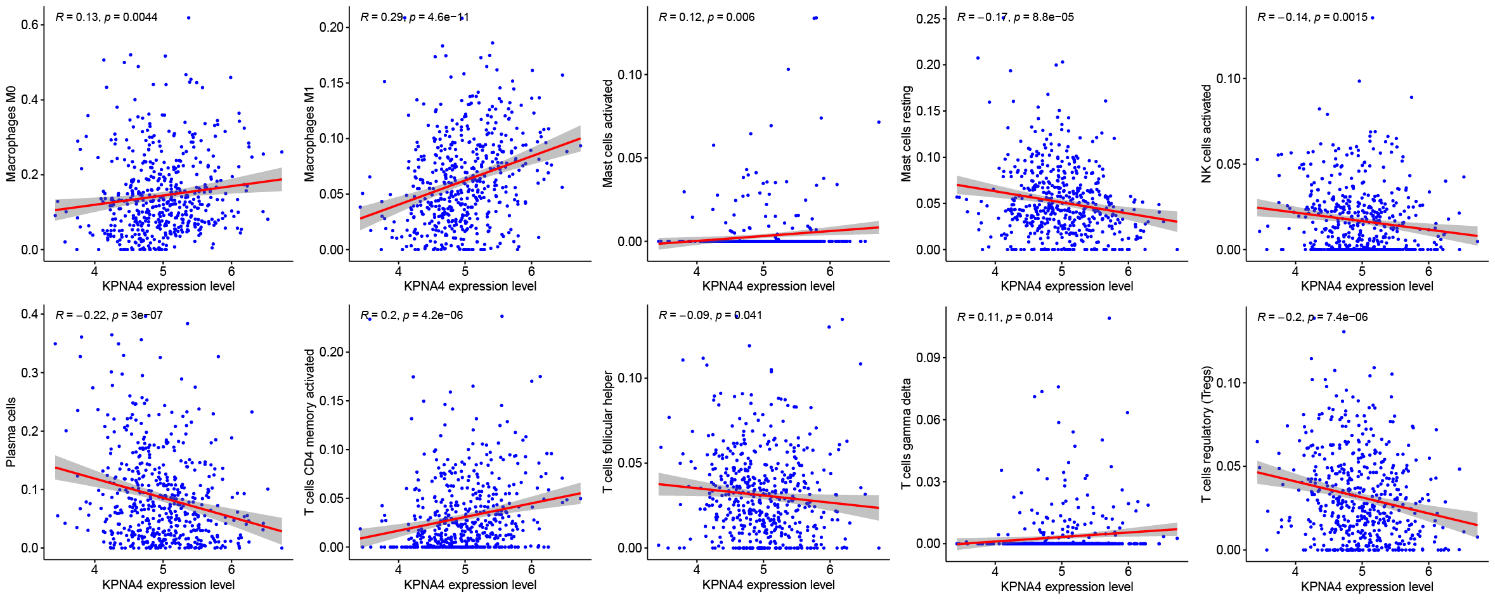


**Supplementary Figure 3.** Correlation between KPNA4 and immune cells.


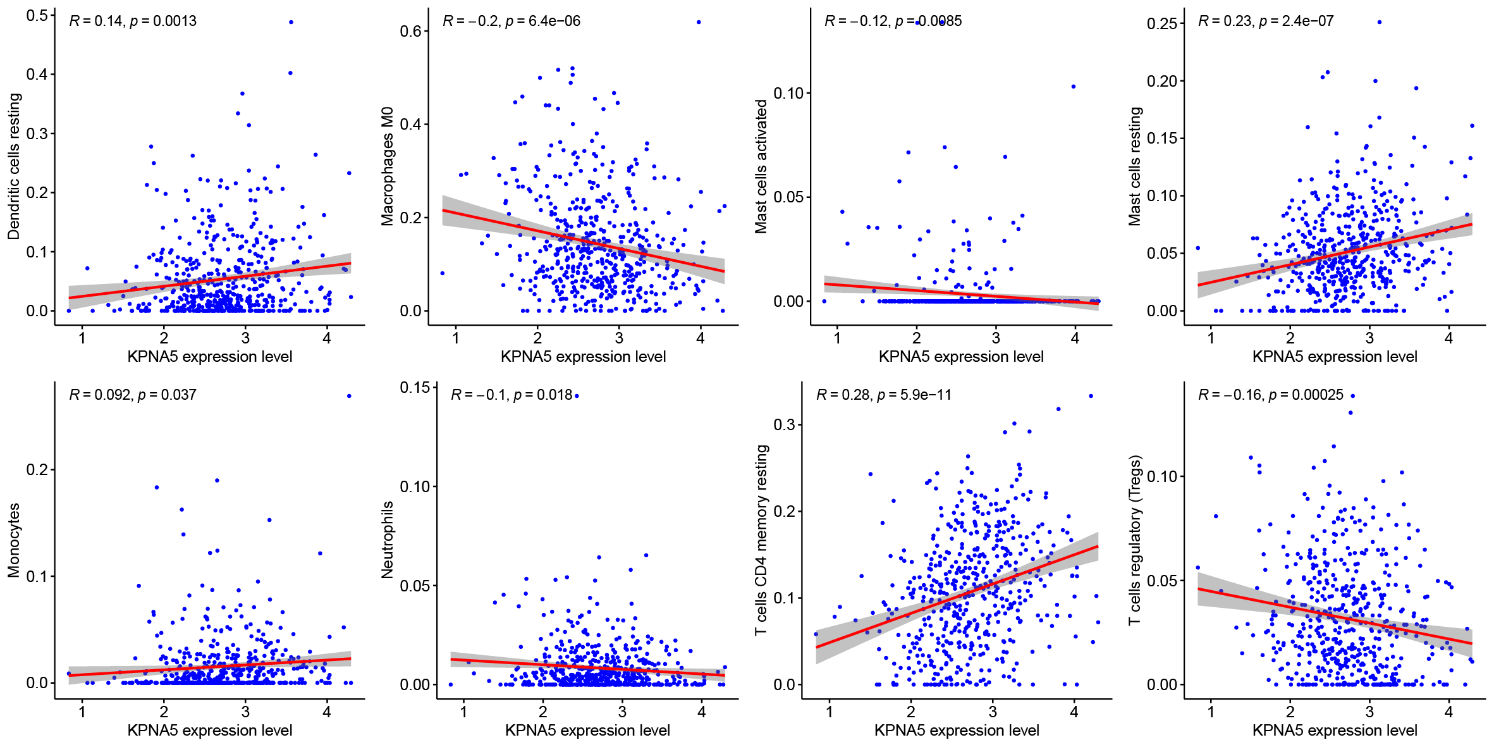


**Supplementary Figure 4.** Correlation between KPNA5 and immune cells.

## Supplementary Tables

**Supplementary Table 1. KPNA family information**

| **Symbol** | **Chr Locus** | **Gene ID** | **Accession** |
| --- | --- | --- | --- |
| KPNA1 | 3q21 | 3836 | NM_002264.3 |
| KPNA2 | 17q24 | 3838 | NM_002266.2 |
| KPNA3 | 13q14 | 3839 | NM_002267.3 |
| KPNA4 | 3q25 | 3840 | NM_002268.3 |
| KPNA5 | 6q22 | 3841 | NM_002269.2 |
| KPNA6 | 1p35 | 23633 | NM_012316.4 |
| KPNA7 | 7q22 | 402569 | NM_001145715.1 |

**Supplementary Table 2. Gene ontology (GO) enrichment analysis**

| **Class** | **ID** | **Descrption** | **num** | **Pvalue** |
| --- | --- | --- | --- | --- |
| Biological Process | GO:0070268 | cornification | 10 | 1.27E-08 |
| Biological Process | GO:0009888 | tissue development | 35 | 2.93E-06 |
| Biological Process | GO:0060429 | epithelium development | 26 | 4.90E-06 |
| Biological Process | GO:0031424 | keratinization | 10 | 8.15E-06 |
| Biological Process | GO:0061138 | morphogenesis of a branching epithelium | 9 | 9.58E-06 |
| Biological Process | GO:0001763 | morphogenesis of a branching structure | 9 | 1.74E-05 |
| Biological Process | GO:0010459 | negative regulation of heart rate | 3 | 8.82E-05 |
| Biological Process | GO:0030216 | keratinocyte differentiation | 10 | 1.05E-04 |
| Biological Process | GO:0019730 | antimicrobial humoral response | 7 | 1.11E-04 |
| Biological Process | GO:0097377 | spinal cord interneuron axon guidance | 2 | 1.69E-04 |
| Biological Process | GO:0097376 | interneuron axon guidance | 2 | 3.36E-04 |
| Biological Process | GO:0061844 | antimicrobial humoral immune response mediated by antimicrobial peptide | 5 | 3.69E-04 |
| Biological Process | GO:0009913 | epidermal cell differentiation | 10 | 4.87E-04 |
| Biological Process | GO:0043576 | regulation of respiratory gaseous exchange | 3 | 5.84E-04 |
| Biological Process | GO:0098900 | regulation of action potential | 4 | 7.34E-04 |
| Biological Process | GO:0045822 | negative regulation of heart contraction | 3 | 7.59E-04 |
| Biological Process | GO:0097065 | anterior head development | 2 | 8.33E-04 |
| Biological Process | GO:0030855 | epithelial cell differentiation | 15 | 8.93E-04 |
| Biological Process | GO:0002027 | regulation of heart rate | 5 | 9.67E-04 |
| Biological Process | GO:0008544 | epidermis development | 11 | 9.80E-04 |
| Cellular Component | GO:0005576 | extracellular region | 57 | 1.77E-05 |
| Cellular Component | GO:0001533 | cornified envelope | 5 | 2.45E-05 |
| Cellular Component | GO:0000785 | chromatin | 19 | 3.88E-03 |
| Cellular Component | GO:0036157 | outer dynein arm | 2 | 3.90E-03 |
| Cellular Component | GO:0045111 | intermediate filament cytoskeleton | 7 | 4.70E-03 |
| Cellular Component | GO:0044447 | axoneme part | 3 | 6.10E-03 |
| Cellular Component | GO:0045095 | keratin filament | 4 | 7.65E-03 |
| Cellular Component | GO:0005585 | collagen type II trimer | 1 | 7.91E-03 |
| Cellular Component | GO:0120135 | distal portion of axoneme | 1 | 7.91E-03 |
| Cellular Component | GO:0150034 | distal axon | 7 | 1.12E-02 |
| Cellular Component | GO:0005858 | axonemal dynein complex | 2 | 1.53E-02 |
| Cellular Component | GO:0070554 | synaptobrevin 2-SNAP-25-syntaxin-3-complexin complex | 1 | 1.58E-02 |
| Cellular Component | GO:0045178 | basal part of cell | 3 | 1.61E-02 |
| Cellular Component | GO:0030057 | desmosome | 2 | 1.66E-02 |
| Cellular Component | GO:0005615 | extracellular space | 37 | 1.77E-02 |
| Cellular Component | GO:0043679 | axon terminus | 4 | 1.89E-02 |
| Cellular Component | GO:0032039 | integrator complex | 2 | 2.05E-02 |
| Cellular Component | GO:0010494 | cytoplasmic stress granule | 3 | 2.32E-02 |
| Cellular Component | GO:0035985 | senescence-associated heterochromatin focus | 1 | 2.36E-02 |
| Cellular Component | GO:0070033 | synaptobrevin 2-SNAP-25-syntaxin-1a-complexin II complex | 1 | 2.36E-02 |
| Molecular Function | GO:0000981 | DNA-binding transcription factor activity, RNA polymerase II-specific | 22 | 1.18E-03 |
| Molecular Function | GO:0005343 | organic acid:sodium symporter activity | 3 | 1.41E-03 |
| Molecular Function | GO:1990837 | sequence-specific double-stranded DNA binding | 24 | 1.42E-03 |
| Molecular Function | GO:0000977 | RNA polymerase II regulatory region sequence-specific DNA binding | 22 | 1.71E-03 |
| Molecular Function | GO:0001012 | RNA polymerase II regulatory region DNA binding | 22 | 1.71E-03 |
| Molecular Function | GO:0003700 | DNA-binding transcription factor activity | 22 | 2.01E-03 |
| Molecular Function | GO:0071855 | neuropeptide receptor binding | 3 | 2.71E-03 |
| Molecular Function | GO:0003690 | double-stranded DNA binding | 24 | 3.04E-03 |
| Molecular Function | GO:0043565 | sequence-specific DNA binding | 24 | 3.24E-03 |
| Molecular Function | GO:0005179 | hormone activity | 5 | 3.54E-03 |
| Molecular Function | GO:0000976 | transcription regulatory region sequence-specific DNA binding | 22 | 3.82E-03 |
| Molecular Function | GO:0044212 | transcription regulatory region DNA binding | 22 | 3.92E-03 |
| Molecular Function | GO:0001067 | regulatory region nucleic acid binding | 22 | 3.98E-03 |
| Molecular Function | GO:0005283 | amino acid:sodium symporter activity | 2 | 6.21E-03 |
| Molecular Function | GO:0030280 | structural constituent of epidermis | 2 | 6.21E-03 |
| Molecular Function | GO:0004867 | serine-type endopeptidase inhibitor activity | 4 | 7.90E-03 |
| Molecular Function | GO:0035501 | MH1 domain binding | 1 | 7.99E-03 |
| Molecular Function | GO:0070009 | serine-type aminopeptidase activity | 1 | 7.99E-03 |
| Molecular Function | GO:0097160 | polychlorinated biphenyl binding | 1 | 7.99E-03 |
| Molecular Function | GO:0005326 | neurotransmitter transporter activity | 3 | 9.19E-03 |

**Supplementary Table 3. KEGG enrichment analysis**

| **KEGG ID** | **Description** | **class** | **Pvalue** |
| --- | --- | --- | --- |
| ko04080 | Neuroactive ligand-receptor interaction | Environmental Information Processing | 3.37E-05 |
| ko04970 | Salivary secretion | Organismal Systems | 9.93E-03 |
| ko00052 | Galactose metabolism | Metabolism | 1.09E-02 |
| ko04270 | Vascular smooth muscle contraction | Organismal Systems | 2.67E-02 |
| ko04915 | Estrogen signaling pathway | Organismal Systems | 2.99E-02 |
| ko05202 | Transcriptional misregulation in cancers | Human Diseases | 3.89E-02 |
| ko04020 | Calcium signaling pathway | Environmental Information Processing | 4.12E-02 |
| ko04924 | Renin secretion | Organismal Systems | 4.79E-02 |

**Supplementary Table 4. Gene set enrichment analysis (GSEA)**

|  | **Description** | **setSize** | **NES** | ***P*.adjust** |
| --- | --- | --- | --- | --- |
| Top 10 in high-risk group | REACTOME_CELL_CYCLE_CHECKPOINTS | 248 | 2.85 | 2.05E-02 |
|  | REACTOME_CELL_CYCLE_MITOTIC | 490 | 2.80 | 2.07E-02 |
|  | WP_RETINOBLASTOMA_GENE_IN_CANCER | 87 | 2.73 | 2.05E-02 |
|  | REACTOME_MITOTIC_METAPHASE_AND_ANAPHASE | 228 | 2.64 | 2.05E-02 |
|  | REACTOME_G2_M_CHECKPOINTS | 129 | 2.61 | 2.05E-02 |
|  | REACTOME_M_PHASE | 346 | 2.60 | 2.05E-02 |
|  | REACTOME_DNA_REPLICATION | 127 | 2.59 | 2.05E-02 |
|  | REACTOME_MITOTIC_G1_PHASE_AND_G1_S_TRANSITION | 148 | 2.59 | 2.05E-02 |
|  | REACTOME_S_PHASE | 160 | 2.58 | 2.05E-02 |
|  | REACTOME_FORMATION_OF_THE_CORNIFIED_ENVELOPE | 129 | 2.58 | 2.05E-02 |
| Top 10 in high low group | KEGG_INTESTINAL_IMMUNE_NETWORK_FOR_IGA_PRODUCTION | 46 | -2.02 | 2.05E-02 |
|  | REACTOME_ROLE_OF_LAT2_NTAL_LAB_ON_CALCIUM_MOBILIZATION | 71 | -2.05 | 2.05E-02 |
|  | REACTOME_FCERI_MEDIATED_CA_2_MOBILIZATION | 86 | -2.06 | 2.05E-02 |
|  | REACTOME_SURFACTANT_METABOLISM | 28 | -2.07 | 2.05E-02 |
|  | REACTOME_CREATION_OF_C4_AND_C2_ACTIVATORS | 71 | -2.07 | 2.05E-02 |
|  | REACTOME_INITIAL_TRIGGERING_OF_COMPLEMENT | 79 | -2.14 | 2.05E-02 |
|  | REACTOME_ANTIGEN_ACTIVATES_B_CELL_RECEPTOR_BCR_LEADING_TO_GENERATION_OF_SECOND_MESSENGERS | 86 | -2.15 | 2.05E-02 |
|  | KEGG_ASTHMA | 28 | -2.15 | 2.05E-02 |
|  | REACTOME_SCAVENGING_OF_HEME_FROM_PLASMA | 68 | -2.17 | 2.05E-02 |
|  | REACTOME_CD22_MEDIATED_BCR_REGULATION | 61 | -2.23 | 2.05E-02 |

**
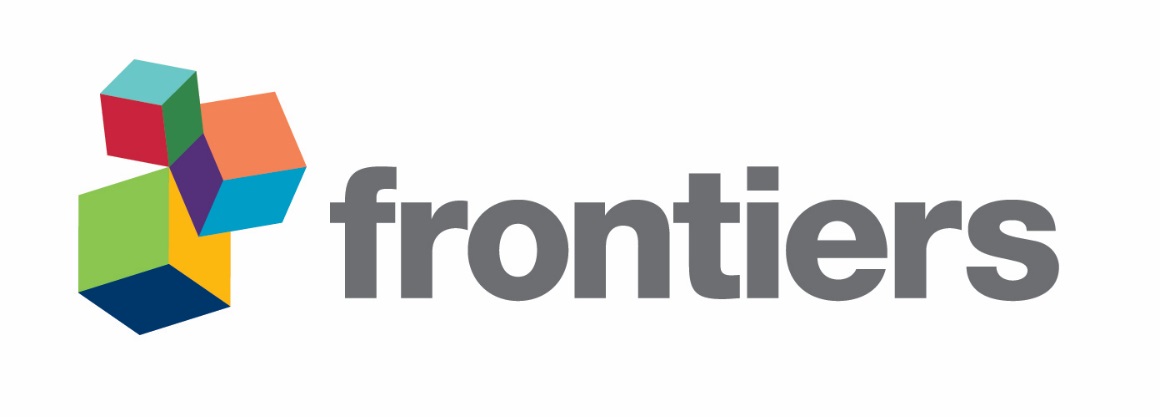
**
